# Lightweight and efficient dual-path fusion network for iris segmentation

**DOI:** 10.1038/s41598-023-39743-w

**Published:** 2023-08-28

**Authors:** Songze Lei, Aokui Shan, Bo Liu, Yanxiao Zhao, Wei Xiang

**Affiliations:** 1 School of Computer Science and Engineering, Xi’an Technological University, Xi’an, Shaanxi China; 2 School of Information Communication Engineering, Beijing Information Science and Technology University, Beijing, China; 3 School of Computing Engineering and Mathematical Sciences, La Trobe University, VIC 3086 Melbourne, Australia; 4 College of Science and Engineering, James Cook University, Cairns, QLD 4878 Australia

**Keywords:** Image processing, Machine learning

## Abstract

In order to tackle limitations of current iris segmentation methods based on deep learning, such as an enormous amount of parameters, intensive computation and excessive storage space, a lightweight and efficient iris segmentation network is proposed in this article. Based on the classical semantic segmentation network U-net, the proposed approach designs a dual-path fusion network model to integrate deep semantic information and rich shallow context information at multiple levels. Our model uses the depth-wise separable convolution for feature extraction and introduces a novel attention mechanism, which strengthens the capability of extracting significant features as well as the segmentation capability of the network. Experiments on four public datasets reveal that the proposed approach can raise the MIoU and F1 scores by 15% and 9% on average compared with traditional methods, respectively, and 1.5% and 2.5% on average compared with the classical semantic segmentation method U-net and other relevant methods. Compared with the U-net, the proposed approach reduces about 80%, 90% and 99% in terms of computation, parameters and storage, respectively, and the average run time up to 0.02 s. Our approach not only exhibits a good performance, but also is simpler in terms of computation, parameters and storage compared with existing classical semantic segmentation methods.

## Introduction

With the development of technology, safety and privacy topics become progressively significant, and identity authentication is widely considered as a concern. Biometric technology is commonly used in various identity authentication scenarios. Biometrics has the characteristics of convenience, universality, security, uniqueness, etc.^1^. Among various biometrics, iris recognition is considered the most prospective identity recognition approach due to its stabilization, reliability and contactless nature of the recognition procedure. A whole iris recognition procedure consists of iris picture acquisition, image preparation, iris segmentation, iris feature extraction, and iris comparison, in which iris segmentation plays a crucial part and has a large effect on the iris recognition's precision.

Traditional iris segmentation approaches consist of two major classes, namely border-based approaches and pixel-based approaches. The first type of methods obtains the complete and independent iris region by obtaining the segmentation boundaries of the iris, the position of the topmost and lower eyelids, and reducing the effects of eyelash occlusion and mirror refraction. This type of methods can achieve good segmentation results on clear iris images under ideal conditions, but the segmentation is not satisfactory when dealing with iris images obtained at long distances and under visible light. These methods usually require manual feature design, and feature selection and training of classifiers are performed separately. Consequently, they face significant challenges in dealing with iris segmentation in complex scenes.

Recently, along with the continuous increased of computer computing capability and the application of big data, deep learning approaches have gradually come into the limelight and been increasingly applied to iris segmentation. Iris segmentation methods in the context of deep learning are superior to traditional iris segmentation methods, but they cannot achieve high efficiency and accuracy simultaneously. The large number of participants and the storage space taken up are the issues facing practical iris recognition systems. Given these challenges, a lightweight iris segmentation network is proposed in this paper, which uses the U-net as the backbone framework and has a parallel branch with an attention mechanism and a feature fusion module. The main contributions are three-fold in this article:

1. A dual-path fusion network structure is proposed. Parallel branches are designed to extract shallow spatial features into the main network and to fuse shallow spatial information with deep semantic information in an attempt to improve the performance of the network as well as segmentation accuracy.
2. We introduce a novel attention mechanism, which embeds position details into the channel attention by encoding the feature map along two spatial directions. Then, it captures the global receptive field and encodes the accurate position information simultaneously. In this way, the weight of the iris region is increased, and the effect due to unrelated information and noise is reduced.
3. We design a lightweight network, which can decrease the quantities of both network parameters and computation, while assuring the network capability and segmentation precision.

## Related work

The current mainstream research directions on iris segmentation are divided into traditional methods based on the supposition that the inside and outside circles of iris are circular, and deep learning-based segmentation approaches. Traditional iris segmentation approaches mainly include Daugman^2^ and Wildes^3^. Both methods assume that the interior and exterior borders of the iris are circular and that the variance in pixel gray values at the iris border is large. There are many subsequent and improved works based on these two types of methods. He et al*.*^4^ proposed a series of robust operations to implement iris segmentation, Tan Tieniu et al.^5^ proposed a coarse iris localization approach based on clustering and noisy region detection and improved the integral differential operator method, and Sutra et al.^6^ used the Viterbi algorithm for iris segmentation. Traditional iris segmentation methods entail a lot of preprocessing and manual operations, which inevitably have a significant impact on the precision and thus affect the quality of iris segmentation results.

Recently, deep learning technology has been applied to iris segmentation’s field, and the problem of iris segmentation is concerned mainly with the development of semantic segmentation networks. Long et al.^7^ first applied the Fully-connected Convolutional Network (FCN) to pixel-level image segmentation based on the CNN, and then various semantic segmentation networks have emerged. In response to the problems of the fixed perceptual field and easy loss or smoothing of segmented object details during semantic segmentation, SegNet^8^ was propose that the downsampling is made up of convolutional layers and pooling layers of VGG16 network, the corresponding upsampling, and the final classification results are yielded by Softmax. The U-Net^9^ has been extensively applied in medical imaging segmentation with a U-shaped symmetric structure consisting of an encoder–decoder pair. The encoder is used to obtain context related information, while the decoder is used to precisely locate the segmentation boundary. The DeepLab^10^ proposes a new dilated convolutional semantic segmentation network that utilizes the spatial coherence between pixels and thus can increase the perceptual field without increasing the quantity of parameters. The researchers introduced the semantic segmentation network into the iris segmentation problem based on the fact that iris segmentation is a binary semantic segmentation problem. Gangwar et al.^11^ proposed a dual-structure network based on the CNN dubbed the iris boundary detection network and segmentation network, which offers a good performance under non-ideal conditions. Wang et al.^12^ proposed a deep multitask learning framework IrisParseNet to improve the performance of iris segmentation and localization by using the intrinsic correlation between the pupil, iris and sclera. Chen et al.^13^ proposed a DFCN network combining the FCN and dense block, and achieved F1 scores of 0. 9828, 0. 9812 and 0. 9606 on the CASIA-Iris-interval, IITD and UBIRIS. v2 datasets, respectively, with a network model size of 138. 91 MB.

We also report on some recent works on lightweight models. Zhou et al.^14^ improved on the U-net by proposing the PI-Unet, which is a network structure capable of heterogeneous iris segmentation. The MIoU scores achieved on the CASIA-Iris-interval and UBIRIS. v2 were 97. 50% and 95. 95%, respectively. Zhang et al.^15^ proposed the FD-Unet for iris segmentation by combining the U-Net structure and null convolution, and severally achieve F1 scores of 0. 9481 and 0. 9736 on the UBIRIS. v2 and CASIA-Iris-interval dataset. Wang and Wang^16^ proposed a new lightweight deep neural network based on the CNN, which provides an end-to-end iris segmentation solution and can be integrated into any conventional iris recognition system.

## Proposed approach

### Overall network structure

**Figure 1:**
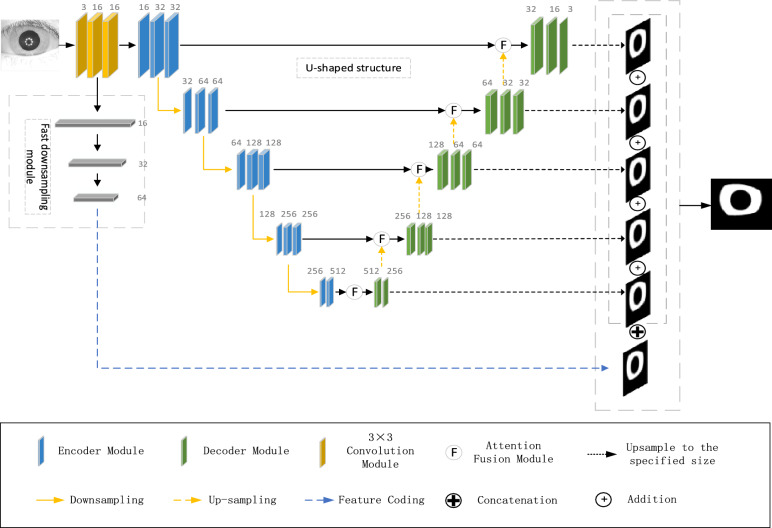
Overall network architecture.

The main structure of the proposed iris segmentation model is divided into two paths as illustrated in Fig. 1. The first path is improved based on the U-net and comprises of an encoder and a decoder. The purpose of the encoder is to extract features of iris image, which includes feature details such as the position and texture. The purpose of the decoder is to switch the iris feature details acquired by the down-sampling module into iris semantic details. The second path is the fast down-sampling module, which aims to obtain richer spatial location information.

An input iris image is first fed into the network through a 3 × 3 convolution module, where the size of the convolution filter is 16 and the stride is 1. The purpose is to expand the number of channels of the image for initial extraction of the iris features without changing the image size, and then output the feature map to both the encoder and the fast down-sampling module.

Unlike the classical U-net, we use depth-separable convolution (Des-convolution)^17^ to substitute the convolution and pooling layers in the encoder. Des-convolution has a smaller number of parameters and faster computation compared to normal convolution, and can make the network deeper with the same number of parameters. The Des-convolution with a stride of two is used to replace the pooling operation in the encoder, which can reduce the loss of information such as pose and space in the image caused by the pooling operation. The down-sampling module is divided into five modules, each of which consists of 3 layers of Des-convolution and has a convolution filter size of 3. Except the first layer, which uses a step-2 convolution for down-sampling, the rest are all step-1 convolutions, followed by down-sampling to ensure the accuracy of segmentation with reduced parameters and lower computation. The up-sampling component is also made up of five modules with each module consisting of a deconvolution and a Des-convolution, where the Des-convolution filter size is 3. The feature image is up-sampled to the primary size by the deconvolution and Des-convolution, and the feature image of each stage of the decoder is up-sampled to the same resolution of the feature map of the corresponding stage of the encoder, and the feature converge is carried out by means of concatenation. This effectively enhances the feature capture capacity of the network and avoid the matter of sharp gradient fluctuations and consequent degradation of model performance.

The second path in the fast down-sampling module consists of three layers of depth-separable convolutions, in which the filter size is 3 × 3 and the stride is 2. Fast down-sampling is performed on 1/8 of the original image, and this module can encode rich low-level spatial and detail information. The first path is deep enough to have a large perceptual field, and the network output is high-level contextual information. The outputs of the two paths are merged in the final stage of the network, and the segmented images are finally classified by the Sigmoid activation function.

### Feature fusion part

The feature fusion part in this paper is represented by two structures in the network, namely the attention-weighted U-shaped path fusion module and the dual paths fusion module.

#### Attention-weighted U-shaped path fusion

When an input iris image is fed to the network for feature extraction, some useless location or spatial information may be retained, which in turn affects the precision of iris segmentation. To tackle the problem, the attention mechanism is introduced. In the attention mechanism, the important information of iris image is enlarged and useless information is suppressed.

The attention mechanisms are divided into diverse categories of channel, spatial, and mixed domain attention mechanisms. Channel attention encodes the nonlinear relationship between channels through convolutions to obtain the weights of each channel and then weights the feature map, which makes the model more capable of discriminating the features of each channel and thus can prominently raise the model performance. However, the disadvantage is that it usually ignores location details, which is crucial for spatially selective weighted maps generation. Spatial attention introduces spatial information encoding to exploit location information by reducing the number of channels and using large-sized convolutions for feature encoding. However, the convolution operation is only able to acquire local correlations and cannot model long-term dependencies of the downstream visual task.

**Figure 2:**
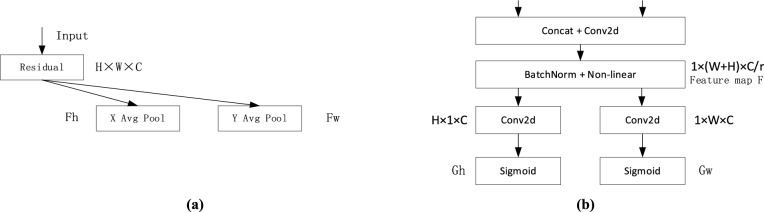
(**a**) Coordinate information embedding. (**b**) Coordinate attention generation.

To address the above issues, the CA (coordinate attention) mechanism is introduced^18^. The CA attention mechanism adopts a more effective method to capture the correlation between spatial location information and channels to enhance the expressiveness of feature maps in neural networks. As shown in Fig. 2a,b, the CA encoding phase is repartitioned into two stages, namely embedding coordinate information and generating coordinate attention. In the first step, as seen in Fig. 2a, a feature map of size H × W × C is the input, and each channel is first encoded towards the horizontal and vertical directions using pooling layers of kernel sizes of (H, 1) and (1, W), respectively, so as to obtain a pair of direction-aware feature maps *Fh* and *Fw*. This approach enables attention module to catch the channel dependencies in one spatial direction while also preserving the accurate location detail of other different spatial direction. This aids the network precisely localize interest regions.

The second stage is to generate Coordinate Attention. As can be observed from Fig. 2b, a pair of feature vectors generated in the first stage have a good perceptual field and accurate location information, and then are concatenated and subjected to a 1 × 1 convolution, and the dimensionality is reduced to the initial C/r. After that, the feature extracted by batch normalization is input into the sigmoid function to acquire a feature map of 1 × (H + W) × C/r F. The activation function enables the attention layer to have a non-linear mapping capability for encoding spatial information in both horizontal and vertical directions. The feature map F is convolved with a filter of size 1 × 1 in the height H and width W directions. After that, two separate tensor sums of dimensions H × 1 × C and 1 × W × C are obtained, and the weights Gh and Gw in the height and width directions are obtained after the activation function sigmoid. The weight information and the initial stage feature map vector are multiplied and weighted to obtain the feature map in the direction of height H and width W.

**Figure 3:**
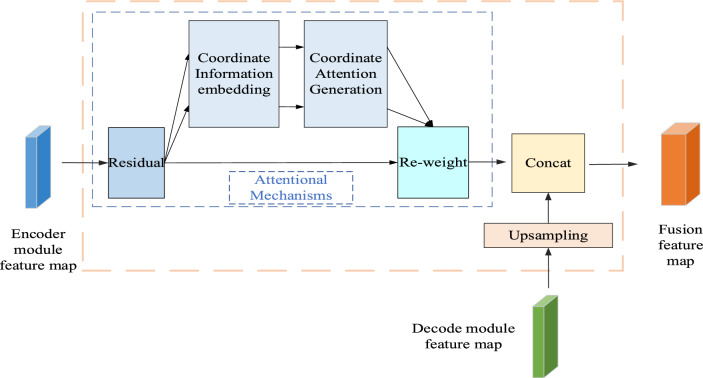
Attention fusion module.

As can be seen from Fig. 3, the attention fusion block is repartitioned into two stages. In first stage, the encoder feature map is weighted. The feature vector of the down-sampling module is fed to the CA module to obtain a weighted feature map, which has two types of representational information, namely spatial location information and inter-channel dependencies. In second stage, the feature image output by the decoder is up-sampled to the identical resolution as the feature map output by the encoder, and then the weighted feature map is concatenated with the up-sampled feature to obtain the fused feature map, which carries more powerful representational information and greatly improves the model segmentation performance. By incorporating the weighted feature map into the up-sampling stage, this operation is more beneficial to convert the feature information of the iris image into iris semantic information, which is beneficial to the final segmentation result.

#### Dual-path fusion

**Figure 4:**
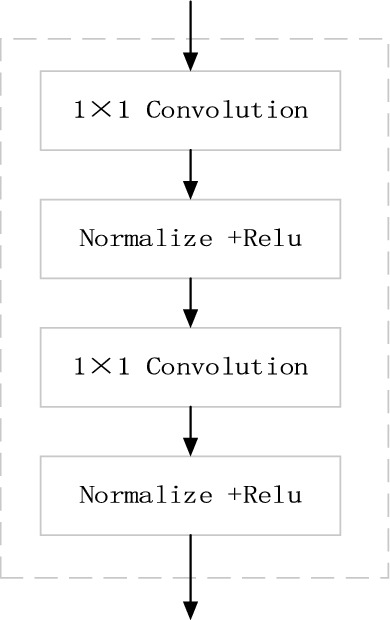
Feature encoding module.

The feature map yielded by the fast downsampling module has rich spatial location information, while the feature map produced by the U-path contains high-level contextual information. Since the two features are mismatched, we propose a feature encoding module. As seen in Fig. 4, this module consists of 1 × 1 convolution, normalization function, and ReLU function, which serves to increase the nonlinear characteristics substantially and improves the representation ability of feature map without altering its structure.

**Figure 5:**
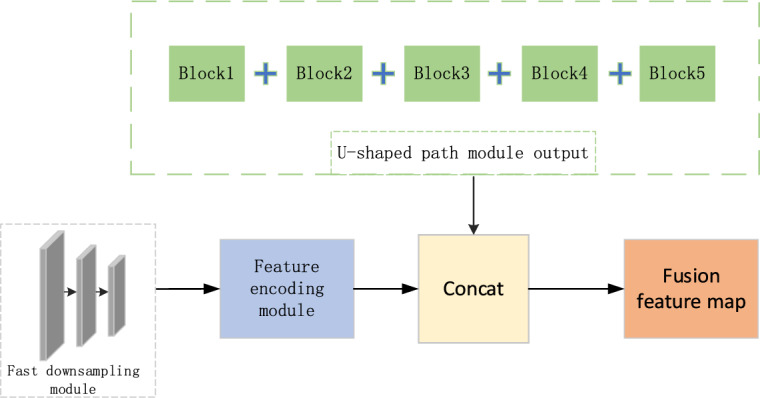
Feature fusion module.

As can be observed for the fusion of dual paths from Fig. 5, the feature maps from the fast down-sampling module are first encoded using the feature encoding module to obtain feature maps with rich spatial location information. Meanwhile, the decoder of the U-path outputs feature maps at every stage that are up-sampled to the same size as the feature maps produced by the feature encoding module, and then these features are summed and normalized to produce a feature map rich in advanced contextual information. Then the characteristic diagram produced by the two paths are concatenated, and the final output feature vector contains both high-level semantic information and low-level spatial position information.

### Loss function

The goal of the loss function is to make the result close to ground-truth label by computing the difference between the network prediction result and actual label, and then the weights yielded from the network training are renewed by backward propagation. For sorting tasks, the network output is the predicted likelihood of each class, followed by how to structure the loss function so that the possible value of the class with positive label is higher.

In this article, the BCE_loss (Binary Cross Entropy) function is added to the network for training, and the loss function is defined as

1 $$loss\left( {z,y} \right) = mean\left\{ {l0,....lN - 1\left. {} \right\}} \right.$$

2 $$ln = - \left( {yn*log\left( {zn} \right) + \left( {1 - yn} \right)*log\left( {1 - zn} \right)} \right)$$

where *n* denotes the number of samples, $zn$ denotes the probability of predicting the *n*-th sample to be a positive case, and $yn$ means the label of the *n*-th sample.

## Experimental results and discussions

### Dataset and data augmentation

**Figure 6:**
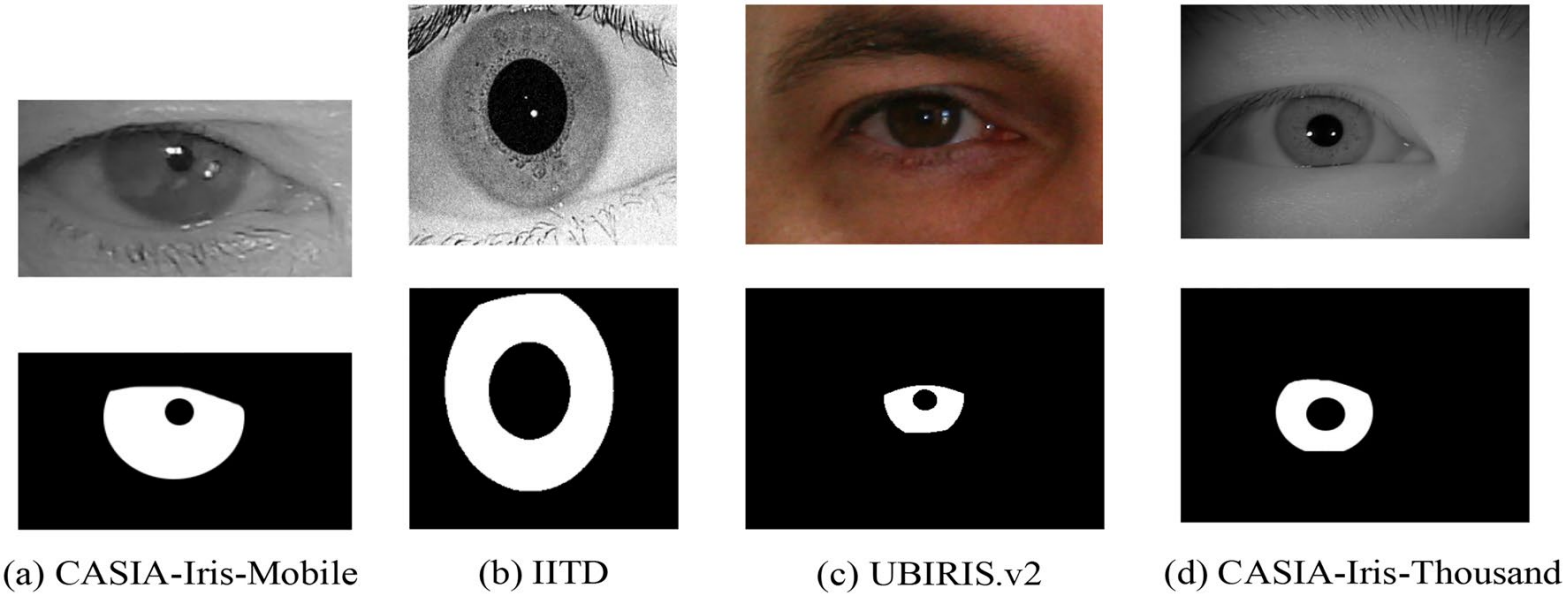
Example datasets.

There are four public datasets used in the experimental studies in this paper, namely CASIA-Iris-Mobile^19^, IITD^20^, CASIA-Iris-Thousand and UBIRIS.V2^21^. CASIA-Iris-Mobile contains totally 11,000 images from 630 Asian subjects. It includes three subsets: CASIA-Iris-M1-S1, CASIA-Iris-M1-S2, and CASIA-Iris-M1-S3. Al mages were collected under NIR illumination and two eyes were captured simultaneously. In this paper we choose the CASIA-Iris-M1-S1 dataset and partial images in the CASIA-Iris-M1-S2 dataset and CASIA-Iris-M1-S3 dataset, which is repartitioned into training set (4000 iris images) and test set (500 iris images) which includes right and left eye images. The IITD iris dataset is provided by the Indian Institute of Technology Delhi and was captured in near-infrared conditions, which includes right and left eye images and the corresponding masked labeled images collected from 224 volunteers. In this paper, this dataset is repartitioned into training set (1120 iris images) and test set (1120 iris images), each of which is of an equal dimension of 224 × 224 pixels. The UBIRIS.V2 dataset is provided by the SOCIA Lab of The University of Beira Interior, which is composed of 261 volunteers’ left and right iris pictures taken at different shooting distances and angles under indoor visible light conditions. In this paper, 2250 iris images of dimension 600 × 800 pixels and their corresponding mask annotations are selected as the dataset, which is partitioned into a training set and a test set at a 3:1 ratio. CASIA-Iris-Thousand contains 20,000 iris images from 1000 subjects. In this paper, this dataset is repartitioned into training set (4000 iris images) and test set (1500 iris images). Each of the above four datasets has unique characteristics due to different specifications, acquisition environments, and image resolutions, which makes it trustworthy to validate the versatility of the proposed network model. Some example images from these datasets and the corresponding mask are shown in Fig. 6.

Due to the insufficient number of available labeled samples, to tackle the problem of overfitting in the proposed network during training, a data augmentation based data processing method is used to improve robustness and to reduce sensitivity to images. In this paper, we use several methods such as flipping, scaling and cropping to extend the dataset and improve the model’s generalization ability. We divide the dataset randomly according to the scaling required, and there may be cases in the experimental phase where the human eye image of the same person appears in both the training dataset and test dataset, and the following experiments are based on the current conditions.

### Training details

The experiments undertaken in this article are based on the PyTorch platform with the following hardware configuration, i.e., the server has 128 GB of running memory and is equipped with four NVIDIA 2080Ti GPUs, each with 12 GB of graphics memory. The model is trained with the Adam optimizer. Initial learning rate is 0.001, the first attenuation rate is set to be 0.9, the second attenuation rate is set to be 0.999, the batch-size is set to be 8, and 200 batches are trained.

### Evaluation indicators

A diverse assessment metrics are used to evaluate the proposed network model to account for performance, efficiency, and accuracy. The assessment metrics employed include the pixel accuracy (PA), Mean Intersection Over Union (MIoU) and F1-score. An iris image is classified at the pixel level, so individual iris pixels can be split into iris regions or non-iris regions. The iris areas are noted as Positive (P) and non-iris regions are noted as Negative (N), the correct output is noted as True (T) and the wrong output is noted as False (F). The classification results are divided into four classes, i.e., True Positive, True Negative, False Positive, and False Negative. The following are formulas for computing the above evaluation metrics:

3 $$MIoU = \frac{{\left[ {\frac{TP}{{TP + FP + FN}} + \frac{TN}{{TN + FP + FN}}} \right]}}{2}$$

4 $$F1 = \frac{2TP}{{2TP + FP + FN}}$$

5 $$PA = \frac{{\left( {TP + TN} \right)}}{TP + TN + FP + FN}$$

The PA, MIoU and F1 scores fall between 0 and 1 in value. The closer the value is to 1, the higher the accuracy and precision.

### Methods comparison

#### Comparison of different methods

Traditional iris segmentation methods such as the Caht^19^, Ifpp^20^ and Wahet^21^, and the proposed approach are evaluated on the CASIA-IRIS-Mobile dataset with near-infrared iris pictures, IITD dataset, CASIA-Iris-Thousand, UBIRIS dataset with visible iris picture. As shown in Table 1, the proposed segmentation approach can significantly improved the PA, MIoU and F1 scores on all the four datasets compared to the traditional iris segmentation methods. The PA, MIOU and F1 scores are 0.9634, 0.9547 and 0.9781 on the CASIA-Iris-Mobile dataset, 0.9840, 0.9601 and 0.9825 on the IITD dataset, and 0.9913, 0.9510 and 0.9960 on the UBIRIS dataset, 0.9863, 0.9621 and 0.9747 on the CASIA-Iris-Thousand dataset, respectively. The DL-based iris segmentation approaches are more accurate and robust.

**Table 1:** Evaluation matrices of the comparison traditional methods on the four datasets.

Iris segmentation method	CASIA Moible PA	CASIA Moible MIoU	CASIA Moible F1	IITD PA	IITD MIoU	IITD F1	UBIRIS PA	UBIRIS MIoU	UBIRIS F1	CASIA Th-d PA	CASIA Th-d MIoU	CASIA Th-d F1
Caht^22^	–	0.7612	0.7314	–	–	–	–	–	0.1048	–	–	–
Ifpp^23^	0.6597	0.7571	–	–	–	–	–	–	0.2899	–	–	–
Wahet^24^	0.7630	0.7745	0.8512	–	–	–	–	–	0.1977	–	–	–
Ahmad^25^	0.8716	–	–	0.9314	–	0.9520	0.8927	–	–	–	–	–
GST^26^	–	–	–	–	–	0.9393	–	–	–	–	–	–
Our approach	0.9634	0.9547	0.9781	0.9840	0.9601	0.9825	0.9913	0.9510	0.9960	0.9851	0.9612	0.9714

As shown in Table 2, deep learning (DL)-based iris segmentation methods such as the Unet^15^, DeepLabV3^27^ and FD-Unet^16^ evaluated on the CASIA-Iris-Mobile dataset, IITD dataset, CASIA-Iris-Thousand with near-infrared iris images and UBIRIS dataset with visible iris images. In comparison with the U-net, the proposed approach also achieves a good performance, improving 0.03 and 0.05 in PA, 0.03 and 0.02 in MIoU, and 0.03 and 0.04 in F1 score on the CASIA-Iris-Mobile and UBIRIS datasets, respectively.

**Table 2:** Evaluation matrices of the comparison DL-based methods on the four datasets.

Iris segmentation method	CASIA Moible PA	CASIA MoibleMIoU	CASIA MoibleF1	IITD PA	IITD MIoU	IITD F1	UBIRIS PA	UBIRIS MIoU	UBIRIS F1 Score	CASIA Th-d PA	CASIA Th-d MIoU	CASIA Th-d F1
Unet^15^	0.9363	0.9219	0.9422	0.9571	0.9501	0.9618	0.9314	0.9362	0.9553	0.9367	0.9374	0.9421
DeepLabV3^27^	0.8633	0.8734	0.8921	0.9161	–	–	0.8947	0.7024	0.8755	0.8867	–	–
FD-Unet^16^	0.9312	–	0.9476	0.9413	–	0.9481	0.9356	–	–	0.9417	–	0.9183
Shufflenet	0.9412	0.9341	0.9521	–	–	–	0.9213	0.9102	0.9142	–	–	–
Mobilenet	0.9321	0.9264	0.9481	–	–	–	0.9341	0.9356	0.9471	0.9421	0.9441	0.9401
Ghostnet^28–30^	–	–	–	0.9541	0.9647	0.9531	–	–	–	0.9402	0.9353	0.9325
Our approach	0.9634	0.9547	0.9781	0.9840	0.9601	0.9825	0.9913	0.9510	0.9960	0.9851	0.9612	0.9714

Table 3 lists the number of parameters, computation (number of floating-point computations per second), and storage space of the DL-based segmentation methods when the input image size is 280 × 280 pixels. The number of network parameters, computation, storage space and running time of the proposed approach are superior to the DL-based segmentation methods of the U-net, DeepLabV3, and Linknet. Compared with lightweight networks such as Shufflenet, Mobilenet and Ghostnet, the proposed network achieves a superior performance. We generally used a simple and directly connected encoder and decoder structure. The Shufflenet, Mobilenet and Ghostnet were used as the backbone of the encoder module and used the same decoder module, which consists of three convolutional layers and activation functions alternately, making the images unsampled to the appropriate scale and finally classified by the softmax layer.

**Table 3:** Number of parameters, computation, storage space, and running time of comparison methods.

Method	Params/M	FLOPs/GMac	Storage space/MB	Average time/s
U-Net^15^	34.53	65.51	517	0.65
DeepLabV3^27^	18.86	–	–	0.44
Linknet^31^	9.82	0.822	35	–
Shufflenet^28^	**0.88**	3.26	**3.56**	0.12
Mobilenet^29^	3.21	3.70	12.72	0.23
Ghostnet^30^	5.18	**0.25**	20.45	0.16
Our approach	1.99	1.16	7.4	**0.02**

#### Experimental results

**Figure 7:**
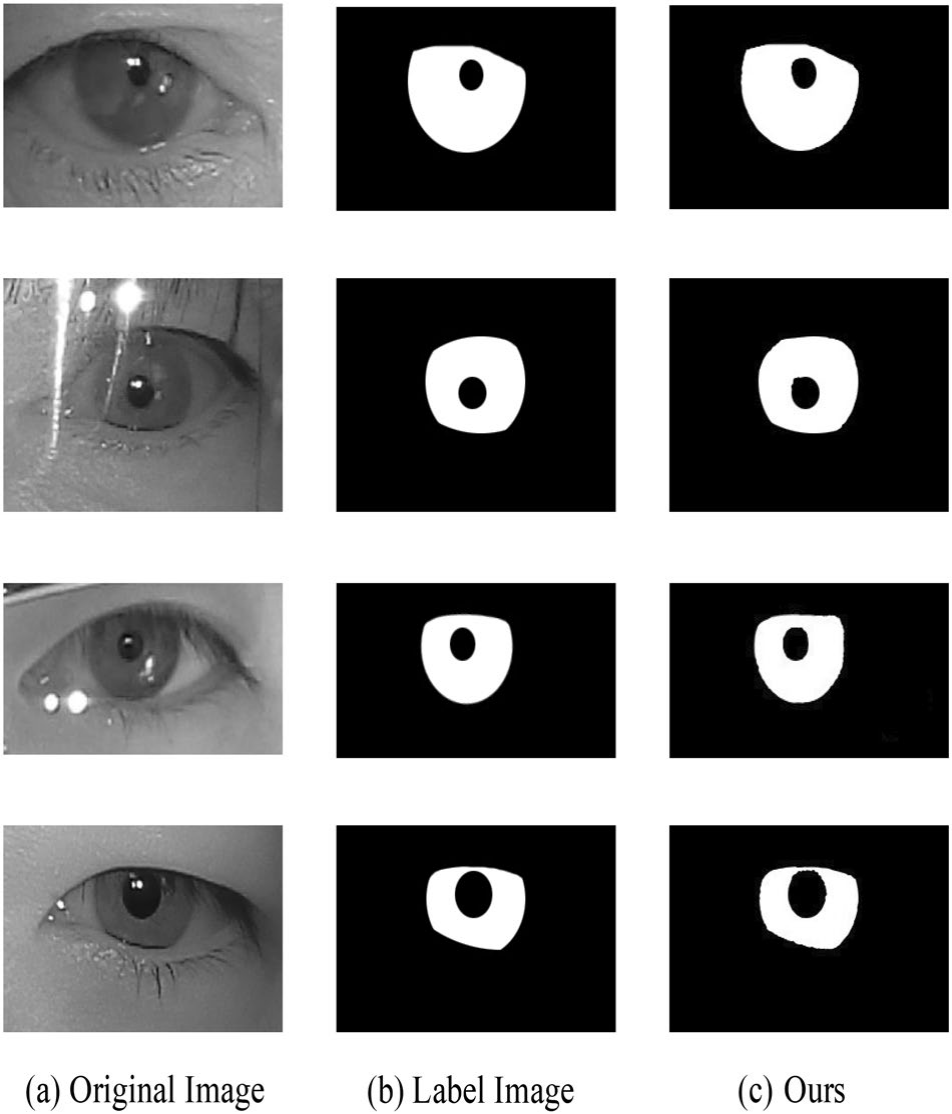
Segmentation results on the CASIA-Iris-Mobile.

**Figure 8:**
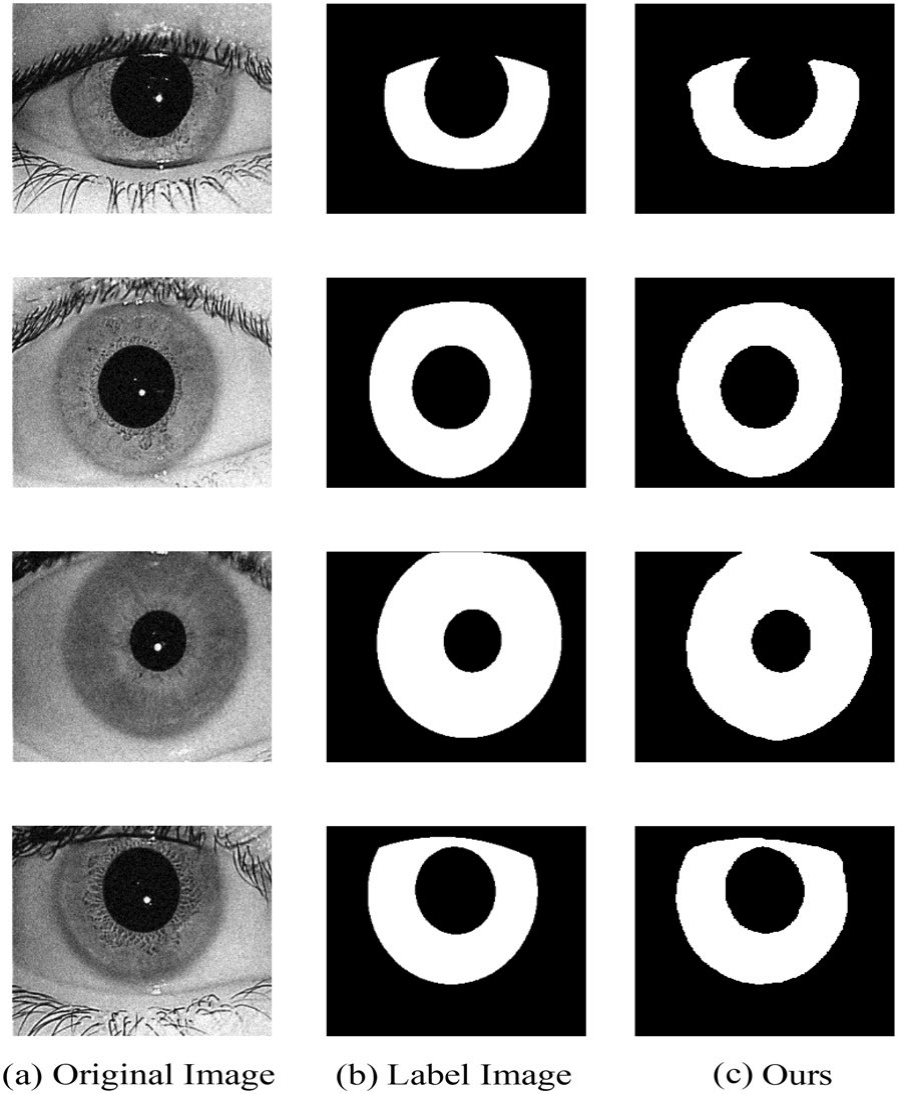
Segmentation results on the IITD.

**Figure 9:**
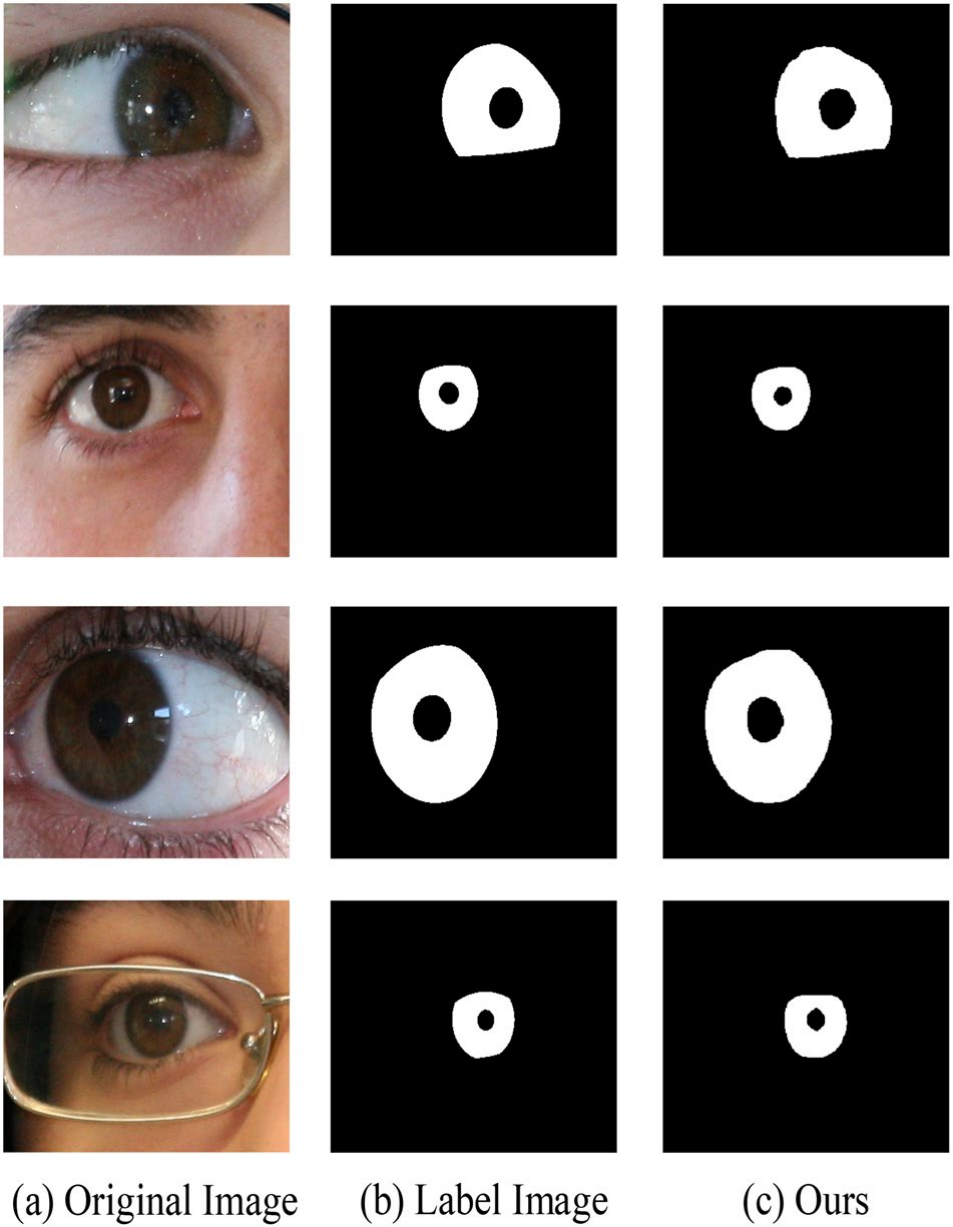
Segmentation results on the UBIRIS v2.

**Figure 10:**
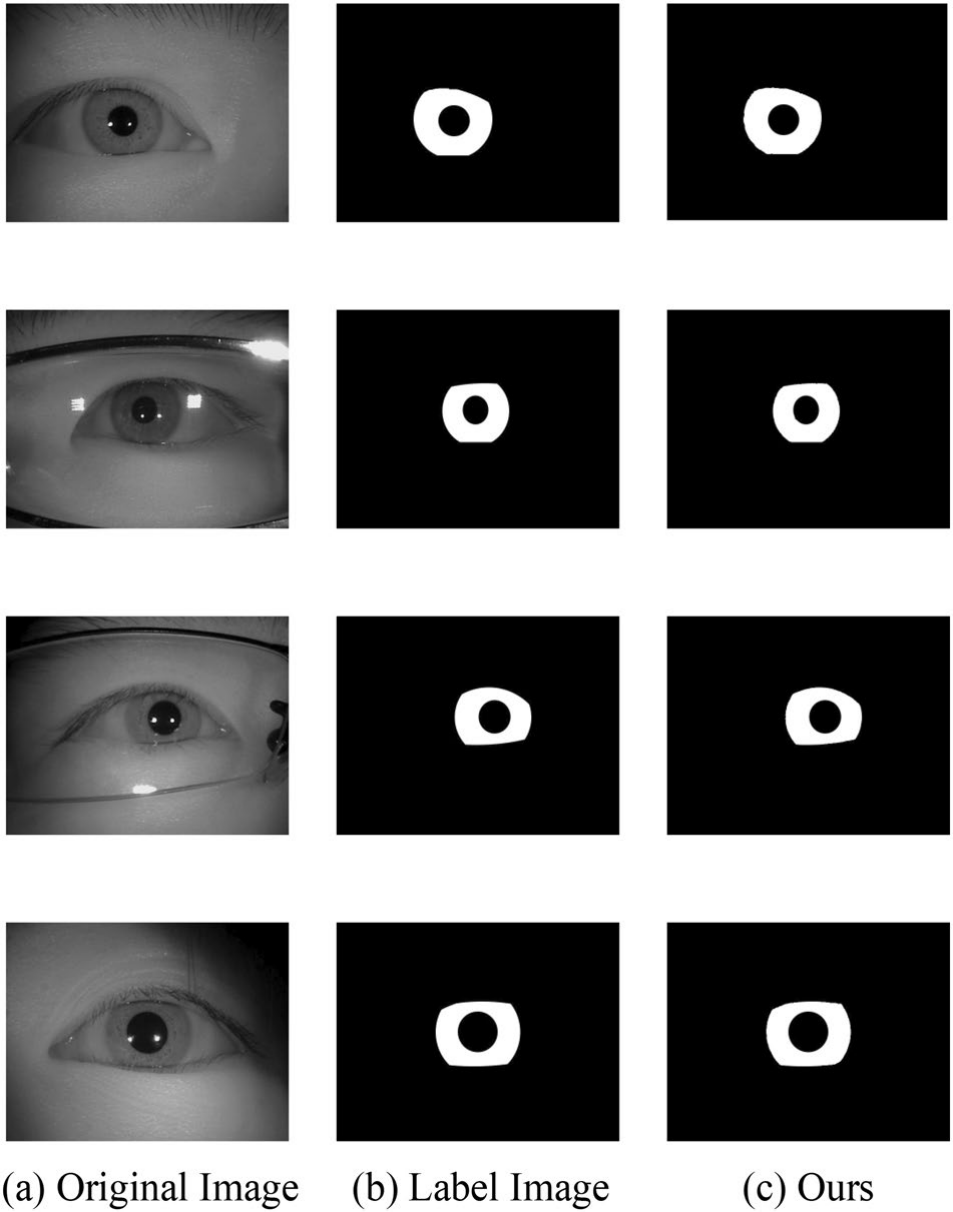
Segmentation results on the CASIA-Iris-Thousand.

In this section, we exhibit the performance of the proposed approach and visualize the iris segmentation results. Figure 7 plots the segmentation results of the proposed approach on the CASIA-Iris-Mobile dataset of near-infrared, Fig. 8 shows the network predict results of the proposed approach on the near-infrared dataset IITD, and Fig. 9 depicts the sorting results of the proposed approach on the UBIRIS dataset under visible light conditions. These experimental results revealed that our proposed approach is able to show superior segmentation results on both the CASIA and IITD datasets due to the high quality of NIR iris images and distinct iris contours. Compared to the UBIRIS dataset, the dataset images have unique characteristics such as out-of-focus blur, contact lens occlusion, and hair occlusion due to its variable imaging distance. It is more challenging for Dl-base segmentation approach, and the proposed approach achieves better segmentation results on this dataset. Figure 10 plots the segmentation results of the proposed approach on the CASIA-Iris-Thousand dataset.

### Ablation experiments

To validate the parallel branch, feature blend block, and the attention mechanism in the proposed network model in enhancing the precision of iris segmentation, four ablation experiments were carried out. The benchmark network used in the experiments is the original U-net using the depth-separable convolution, to which the parallel branch, feature fusion module, and attention mechanism are added to form three different networks. The MIoU and F1 scores of the four networks on the four iris datasets are compared.

As can be observed from the comparison results in Tables 4, 5, 6 and 7, the MIoU scores of our proposed approach on the four datasets are 0.9547, 0.9601, 0.9510 and 0.9721, respectively, corresponding to 1.8%, 1%, 1.5% and 3% improvements against the benchmark network. The experiments also show that the parallel branch, the feature blend block, and the attention mechanism in the proposed network all contribute significant enhancements to the network.

**Table 4:** Results of the ablation experiments on the CASIA-Iris-Mobile.

Parallel branch roads	Feature fusion module	Attention mechanism	MIoU	F1 score
–	–	–	0.9219	0.9422
√	–	–	0.9321	0.9534
√	√	–	0.9476	0.9680
√	√	√	0.9547	0.9781

**Table 5:** Results of the ablation experiments on the IITD.

Parallel branch roads	Feature fusion module	Attention mechanism	MIoU	F1 score
–	–	–	0.9501	0.9618
√	–	–	0.9556	0.9694
√	√	–	0.9583	0.9763
√	√	√	0.9601	0.9825

**Table 6:** Results of the ablation experiments on the UBIRIS.

Parallel branch roads	Feature fusion module	Attention mechanism	MIoU	F1 score
–	–	–	0.9362	0.9553
√	–	–	0.9401	0.9534
√	√	–	0.9455	0.9746
√	√	√	0.9510	0.9960

**Table 7:** Results of the ablation experiments on the CASIA-Iris-Thousand.

Parallel branch roads	Feature fusion module	Attention mechanism	MIoU	F1 score
–	–	–	0.9374	0.9421
√	–	–	0.9401	0.9556
√	√	–	0.9568	0.9679
√	√	√	0.9612	0.9714

**Figure 11:**
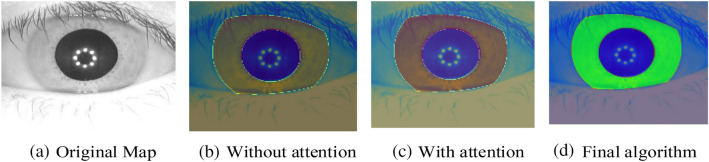
Heat map comparison.

In order to more intuitively demonstrate the ability of the network in extracting the iris feature area and the improvement of the attention mechanism on the network performance, we use heat maps to display the relevant content, as shown in Fig. 10, where Fig. 11a represents the original iris image and Fig. 10b represents the network's perception ability of the iris feature without attention. The light blue area of the iris circumference in the figures represents the irrelevant feature, and the iris area is light yellow. Figure 11c shows the perception ability of the network to the iris features after adding the attention mechanism. Compared with Fig. 11b, the color of the iris region is darker, indicating that the proposed network has a stronger perception ability to the iris region. Figure 11d shows the final perception ability of the network to the characteristics of the iris region, which is shown in green more obviously.

In order to validate the rationality of the network input size chosen in this paper, three different input sizes widely used in the literature were selected for the ablation experiments. The experimental results are shown in Table 8.

**Table 8:** Results of different input size.

Input size	CASIA Mobile PA	CASIA Mobile MIoU	CASIA Mobile F1	IITD PA	IITD MIoU	IITD F1	UBIRIS PA	UBIRIS MIoU	UBIRIS F1	CASIA Th-d PA	CASIA Th-d MIoU	CASIA Th-d F1
224 × 224	0.9378	0.9224	0.9378	0.9321	0.9431	0.9467	0.9531	0.9303	0.9656	0.9421	0.9471	0.9507
320 × 320	0.9541	0.9416	0.9532	0.9565	0.9452	0.9610	0.9712	0.9478	0.9739	0.9701	0.9598	0.9641
280 × 280	0.9634	0.9547	0.9781	0.9840	0.9601	0.9825	0.9913	0.9510	0.9960	0.9851	0.9612	0.9714

According to the experimental results shown in the Table 8, when the input size is 280 × 280, the network performance is the best on all the three datasets.

This paper verifies the universality of the model by cross-training and testing the four datasets. As shown in Table 9 below, the cross-training and testing of the four datasets still offers a good performance.

**Table 9:** Results of cross-training and testing on the four datasets.

Test training	CASIA-Mobile			IITD			UBIRIS			CASIA-Thousand		
	PA	MIOU	F1 score	PA	MIOU	F1 score	PA	MIOU	F1 score	PA	MIOU	F1 score
Mobile	–	–	–	0.9323	0.9116	0.9321	0.9201	0.9341	0.9412	0.9223	0.9124	0.9013
IITD	0.9361	0.9256	0.9401	–	–	–	0.9365	0.9519	0.9561	0.9282	0.9175	0.9012
UBIRIS	0.9441	0.9415	0.9524	0.9652	0.9324	0.9531	–	–	–	0.9374	0.9346	0.9231
Thousand	0.9513	0.9356	0.9354	0.9554	0.9461	0.9353	0.9211	0.9374	0.9364	–	–	–

## Conclusion

Iris segmentation is one of the most critical components of building a trustworthy iris recognition system, and the result of iris segmentation directly affects the precision of iris recognition. The objective of this paper was to enhance both precision and efficiency of iris segmentation. Towards this end, a lightweight iris segmentation network was proposed in this article. The proposed approach adopts the classical semantic segmentation network U-net as the backbone and adds a parallel branch. While the original U-net captures deep contextual information, the parallel branch obtains enough shallow contextual information for fusion. An attention mechanism was introduced to enhance the network's capability to extract important features and to enhance the robustness of the network. Extensive experimental results were presented to reveal that the proposed network model can not only improve performance but also reduce the amount of parameters, computation and storage space compared with existing semantic segmentation methods. It is concluded from the segmentation result images that our proposed approach achieved better segmentation results on the CASIA, IITD, and UBIRIS datasets. Future work will aim to further reduce the quantity of parameters and computation of the model without compromising model accuracy.

## Data Availability

The data that support the findings of this study are available from “the Chinese Academy of Sciences' Institute of Automation (CASIA), Indian Institute of Technology Delhi and SOCIA Lab of The University of Beira Interior” but restrictions apply to the availability of these data, which were used under license for the current study, and so are not publicly available. Data are however available from the authors upon reasonable request and with permission of “the Chinese Academy of Sciences' Institute of Automation (CASIA), Indian Institute of Technology Delhi and SOCIA Lab of The University of Beira Interior”. Departmento de Informática, Universidade da Beira Interior, Covilhaa, Portugal.
